# Hepatic effects of tartrazine (E 102) after systemic exposure are independent of oestrogen receptor interactions in the mouse

**DOI:** 10.1016/j.toxlet.2017.03.024

**Published:** 2017-05-05

**Authors:** Stephanie K. Meyer, Philip M.E. Probert, Anne F. Lakey, Andrew R. Axon, Alistair C. Leitch, Faith M. Williams, Paul A. Jowsey, Peter G. Blain, George E.N. Kass, Matthew C. Wright

**Affiliations:** a Institute Cellular Medicine, Newcastle University, Newcastle Upon Tyne, United Kingdom; b European Food Safety Authority, Via Carlo Magno 1A, 43126 Parma, Italy

**Keywords:** ALP, alkaline phosphatase, ALT, alanine aminotransferase, E2, 17β oestradiol, EtOH, ethanol, hER, human oestrogen receptor, mER, murine oestrogen receptor, OO, olive oil, OSPCA, 5-oxo-1-(4-sulphophenyl)-2-pyrazoline-3-carboxylic acid (a major contaminant of the food additive), PBS, phosphate buffered saline, SA, sulphanilic acid, SA-NAc, sulphanilic acid N-acetate, SCAP, 1-(4-sulphophenyl)-3-carboxy-4-amino-5-pyrazolone, SPH, sulphophenylhydrazine, SSY, sunset yellow, T, Tartrazine, Tg, Tg(NF-κB) mice, w/t, wild type mice, E 102, Liver, NF-κB, Alcohol, oestrogen, Food

## Abstract

Tartrazine is a food colour that activates the transcriptional function of the human oestrogen receptor alpha in an in vitro cell model. Since oestrogens are cholestatic, we hypothesised tartrazine will cause periportal injury to the liver in vivo. To test this hypothesis, tartrazine was initially administered systemically to mice resulting in a periportal recruitment of inflammatory cells, increased serum alkaline phosphatase activity and mild periportal fibrosis. To determine whether an oestrogenic effect may be a key event in this response, tartrazine, sulphonated metabolites and a food additive contaminant were screened for their ability to interact with murine oestrogen receptors. In all cases, there were no interactions as agonists or antagonists and further, no oestrogenicity was observed with tartrazine in an in vivo uterine growth assay. To examine the relevance of the hepatic effects of tartrazine to its use as a food additive, tartrazine was orally administered to transgenic NF-κB-Luc mice. Pre- and concurrent oral treatment with alcohol was incorporated given its potential to promote gut permeability and hepatic inflammation. Tartrazine alone induced NF- κB activities in the colon and liver but there was no periportal recruitment of inflammatory cells or fibrosis. Tartrazine, its sulphonated metabolites and the contaminant inhibited sulphotransferase activities in murine hepatic S9 extracts. Given the role of sulfotransferases in bile acid excretion, the initiating event giving rise to periportal inflammation and subsequent hepatic pathology through systemic tartrazine exposure is therefore potentially associated an inhibition of bile acid sulphation and excretion and not on oestrogen receptor-mediated transcriptional function. However, these effects were restricted to systemic exposures to tartrazine and did not occur to any significant effect after oral exposure.

## Highlights

• Systemic exposure to tartrazine results in hepatic periportal recruitment of inflammatory cells, increased serum alkaline phosphatase activity and mild hepatic periportal fibrosis.
• Tartrazine, its sulphonated metabolites and a common contaminant of the food additive do not interact with murine oestrogen receptors.
• Systemic exposure does not have an oestrogenic effect in mouse in vivo.
• Tartrazine, its sulphonated metabolites and a common contaminant of the food additive inhibited sulphotransferase, which may account for its hepatic effects after systemic exposure.
• The hepatic effects of tartrazine do not occur in mice – with or without co-administration of alcohol – after oral exposure to tartrazine.

## 1 Introduction

Many consumer products including food and personal care items contain endocrine disrupting chemicals (EDCs) which may potentially interfere with the endocrine system in animals and humans (Diamanti-Kandarakis et al., 2009, Zoeller et al., 2012). A large number of EDCs have oestrogenic properties in that they mimic the biological effects of endogenous oestrogens. These chemicals are termed xenoestrogens and they may modulate endogenous oestrogen activity by interfering with endogenous oestrogen signalling or by disrupting synthesis, metabolism and transport of oestrogens (Shanle and Xu, 2011). A common mechanism in modulating oestrogen signalling is through interactions of xenoestrogens with the nuclear oestrogen receptors (ERs), often because they possess structural similarities to endogenous oestrogens (McKenna and O'Malley, 2002).

The ERs belong to the superfamily of steroid hormone nuclear receptors (Tsai and O'Malley, 1994, Hammes and Levin, 2007; see also Nuclear Receptor Signalling Atlas https://www.nursa.org/nursa/index.jsf). Two isoforms of the ER exist; the ERα (Green et al., 1986) and ERβ (Mosselman et al., 1996, Kuiper et al., 1996, Moore et al., 1998). Both ER isoforms are ligand-activated by oestrogens such as endogenous 17β-estradiol (E2) and mediate ER-regulated changes in gene expression by interacting with specific DNA sequences (EREs) (Tsai and O'Malley, 1994, Hammes and Levin, 2007). In order to screen for chemicals having agonistic or antagonistic oestrogenic activity, a human-based reporter gene assay was developed (Axon et al., 2012). Employing this assay, the food colour tartrazine (also known as E 102) was identified as an activator of the human ERα in vitro (Datta and Lundin-Schiller, 2008, Axon et al., 2012).

The liver is a hormonal target for oestrogens via ERα (Ahlbory-Dieker et al., 2009) and determines the circulating levels of oestrogens via metabolic conversion of oestrogens to inactive products (Bondesson et al., 2015, Tsuchiya et al., 2005, Ziegler et al., 2015). Thus, significant inhibition of hepatic oestrogen metabolism through liver disease can result in feminisation in men (Burra, 2013). The liver is also a target organ for the toxic effects of high levels of oestrogens. Elevations in circulating oestrogens are hepatotoxic due to a disruption of bile flow and/or alteration in bile constituents (cholestasis) through a potential combination of ERα-dependent suppression of transporter expression (Yamamoto et al., 2006), ERα-dependent stimulation of canalicular transporter endocytic internalization (Barosso et al., 2012) and/or other signalling pathways such as GPR30 (Zucchetti et al., 2014). Cholestasis leads to an accumulation of bile acids in the liver, which is toxic and results in liver cell death (Woolbright and Jaeschke, 2012). In susceptible individuals, the elevations in circulating oestrogens in pregnancy or through use of contraceptives can be sufficient to lead hepatic failure and death in the absence of liver transplantation (Ozkan et al., 2015).

We hypothesised that tartrazine is a mouse ER activator and that if sufficient intact food chemical is absorbed and reaches the liver, it would have a cholestatic effect. We show that systemic exposure to tartrazine through intraperitoneal administration resulted in a pathology consistent with a cholestatic effect (although bile flow was not examined). However, in vitro reporter gene screening assays with all the known mouse ERs indicated that neither tartrazine, its sulphonated metabolites nor a major sulphonated contaminant of the food colour activated or antagonised any of the murine ER receptors. This was supported by an in vivo mouse uterine growth bioassay with tartrazine administered systemically. Oral exposure to tartrazine resulted in gut and hepatic inflammation (based on activation of NF-κB transcriptional function), but there was no evidence for any periportal inflammatory cell recruitment or fibrosis via this route of exposure, and co-exposing with ethanol to increase gut permeability to the food additive, inhibited these effects. Since tartrazine, its 4 sulphonated metabolites and a major sulphonated contaminant of the food additive inhibited dopamine sulphotransferase in a dose-dependent manner in hepatic S9 extracts, the hepatic effects of systemic exposure to tartrazine may be associated with an inhibition of bile acid sulphate conjugation. However, this effect is unlikely to occur after oral exposure to tartrazine.

## 2 Materials and methods

### 2.1 Materials

The mouse cholangiocyte cell line 603B was a gift from Dr Yedidya Saiman, Mount Sinai School of Medicine, New York. The mouse pancreatic epithelial cell line LTPA was originally obtained from the American Type Culture Collection (ATCC, catalogue CRL-2389, Manassas, Virginia). Tartrazine purity of 85% or greater – which meets the EC specifications for its use as a food additive, E2 and ICI182780 were purchased from Sigma (Poole, UK). The tartrazine metabolites sulphanilic acid (SA) [CAS 121-57-3] and 4-sulphopenylhydrazine (SPH) [CAS 98-71-5] and the contaminant 5-oxo-1-(4-sulphophenyl)-2-pyrazoline-3-carboxylic acid (OSPCA) [CAS 118-47-8, permitted at up to 0.5% in tartrazine preparations when used as a food additive according to EC and JECFA specifications] were purchased from Sigma (Poole, UK). The tartrazine metabolites sulphanilic acid N-acetate (SA-NAc) [CAS 121-62-0] and 1-(4-sulphophenyl)-3-carboxy-4-amino-5-pyrazolone (SCAP) [CAS 2508-84-1] were purchased from Santa Cruz Biotechnology (Dallas, Texas, USA) and custom synthesised by An-gene (Hong Kong) respectively. Analytical data for SCAP is provided in Supplementary Fig. 1.

### 2.2 Animal studies

C57Bl/6 wild type (wt) mice were purchased from Charles River (Kent, UK). Transgenic NF-κB-Luciferase (tg) mice (bearing a transgene composed of three NF- B sites from the Ig light chain promoter coupled to the gene encoding firefly luciferase) were originally obtained from Dr Harald Carlsen (Oslo University, Norway) and were generated and genotyped as previously described (Wallace et al., 2010). All animals had free access to food and water and conditions were kept on a 12 h light/12 h dark cycle at 47% relative humidity at 23 °C ± 1 °C. All experiments were performed under a UK Home Office licence with Local Ethics Committee approval.

#### 2.2.1 Systemic exposure to tartrazine in adult mice

To investigate the effects of direct tartrazine exposure, male 12 week old mice were dosed with tartrazine at 50 mg/kg bw/day [dissolved in 137 mM NaCl, 2.7 mM KCl, 10 mM phosphate pH 7.4 (PBS)] via 5 intraperitoneal injections per week for 2 weeks before termination 24 h after the last administration. Control mice were administered with the PBS vehicle alone. Mice were exposed to E2 [prepared in ethanol:olive oil (1:20, v/v)] by intraperitoneal injection at a dose of 0.5 mg/kg bw/day for 3 consecutive days before termination 24 h after the last administration. Control mice were administered with ethanol:olive oil (1:20, v/v)] vehicle alone.

#### 2.2.2 In vivo uterine growth bioassay

To test for mouse ERα activation in vivo, 19 day old female wt mice were treated with oestrogen or potential xenooestrogens by daily intraperitoneal injection on 4 consecutive days. Compounds were prepared in PBS or ethanol:olive oil (1:20, v/v) solvent vehicle with control mice administered solvent vehicle alone. On day 5, mice were culled and uteri removed and relative wet weight determined.

#### 2.2.3 Oral exposure to tartrazine in adult mice, effect of alcohol

To investigate effects of oral tartrazine exposure, male adult wt and tg mice were pre-treated with 3 g ethanol per kg bodyweight from a 20% (v/v) ethanol solution twice daily by oral gavage for 14 days to increase gut permeability and/or alter the gut microbiota (Kirpich et al., 2012, Szabo, 2015). The control group was pre-dosed with 6.32 g dextrose (Sigma) per kg bodyweight from a 0.33 g/ml dextrose solution to control for the calorific content of ethanol. Following the 14-day pre-treatment period, mice were administered 50 mg tartrazine per kg bodyweight from a 2.6 mg/ml stock in either 20% (v/v) ethanol solution or in 0.33 g/ml dextrose solution by oral gavage twice daily for 10 consecutive weeks. Mice in the control groups were dosed with ethanol or dextrose solution alone. Body weights were measured once a week. Tg mice were imaged for inflammation by live in vivo imaging on an IVIS spectrum (Caliper Life Sciences) essentially as previously outlined (Wallace et al., 2010). D-luciferin was obtained from Synchem (Altenburg, Germany).

### 2.3 Cell line culture

603B cells were cultured in low glucose Dulbecco’s Modified Eagles Medium (Sigma, Dorset, UK), supplemented with 10% (v/v) foetal bovine serum (Sigma) and 80 U/ml of penicillin and streptomycin. LTPA cells were cultured in the above medium further supplemented with 0.1 mM non-essential amino acids (Gibco, Life technologies, Paisley, UK) and 1 mM sodium pyruvate (Gibco, Life technologies). All cell lines were maintained in a humidified atmosphere at 37 °C in 5% CO2 in air.

### 2.4 Transfection and reporter gene assays

The mERα, mERβv1 and mERβv2 cDNA sequences were previously cloned and their transcriptional functionality examined in cholangiocyte cell lines (Meyer et al., 2017). Note, that previous work identified that the LTPA cell line combined with the 3×ERE TATA Luc reporter gene construct (originally constructed by Donald McDonnell and obtained via Adgene plasmid # 11354) are optimal for identifying mERα-dependent transcriptional activation whereas the 603B cells coupled with the (ERE)3-pGL3promoter construct is optimal for identifying ERβ-dependent transcriptional activation (Meyer et al., 2017). In all cases, cells were transiently transfected in 24-well plates with 0.25 μg total DNA per well (pcDNA3.1 expression vector encoding the mouse ERα, ERβv1 or ERβv2 proteins essentially as previously described (Meyer et al., 2017), an oestrogen-responsive luciferase reporter gene construct and a control plasmid (RL-TK) encoding the Renilla luciferase protein under the control of a constitutive thymidine kinase promoter to control for transfection efficiency between wells. Cells were transfected with constructs at a ratio of 6:6:1 using Effectene reagent (Qiagen, Manchester, UK), according to the manufacturer’s instructions. Twenty four hours after transfection, cells were, where applicable, pre-treated with the pure ER antagonist ICI182780 for 6 h before being treated with oestrogens or potential xenoestrogens from 1000-fold concentrated stocks in DMSO or PBS. Control cells were treated with 0.1% v/v DMSO or PBS. The human ERβ expression construct pcDNA Flag ERβ originally cloned by Zhang et al. (2010) and was obtained from Adgene (Adgene plasmid # 35562). To examine potential activation of the human ERβ, ER negative HEK293 cells were transiently transfected in 24-well plates with 0.32 μg total DNA per well – pcDNA Flag ERβ, (ERE)_3_-pGL3promoter (Axon et al., 2012) and the RL-TK control plasmid at a ratio of 6:6:1 respectively) using calcium phosphate. Twenty-four hours after transfection, cells were treated with tartrazine, its metabolites and the contaminant from 1000-fold concentrated stocks in DMSO or PBS. Following exposure for 24 h, luciferase activities were determined using a Dual-Glo luciferase assay kit (Promega).

### 2.5 Immunohistochemistry

Livers were fixed in 10% buffered formalin in PBS for 24 h before paraffin embedding and sectioning at 4 μm. Tissue sections were stained with Haematoxylin and Eosin (H&E) or sirius red as previously described (Marek et al., 2005).

### 2.6 Clinical chemistry

Serum samples were prepared from blood by centrifugation for determination of alkaline phosphatase (ALP) serum enzyme levels (Alkaline Phosphatase Assay Kit (Fluorometric), Abcam) according to the manufacturer’s instructions. Alanine aminotransferase (ALT) serum levels were measured as previously described (Probert et al., 2014).

### 2.7 Liver S9 preparation and sulphotransferase assays

Liver S9 extracts were prepared and sulphotransferase assays performed using 35S 3′-phosphoadenosine-5′-phosphosulfate as previously described (Probert et al., 2016).

## 3 Results

### 3.1 Systemic exposure to tartrazine caused a mild cholestatic liver injury in mice

To investigate the effects that direct exposure to tartrazine may have on the liver, tartrazine was administered to mice by intraperitoneal injection at 50 mg/kg bw/day, 6.7 times the current EU ADI (EFSA Panel on Food Additives and Nutrient Sources added to Food (ANS), 2009). Male mice were chosen for this study since the mouse menstrual cycle is short relative to the time over which the study was performed. Since males have a lower, and more consistent, level of circulating oestrogen, it was considered that a study in males would more likely detect any oestrogenic effect of the administered compounds.

**Fig. 1:**
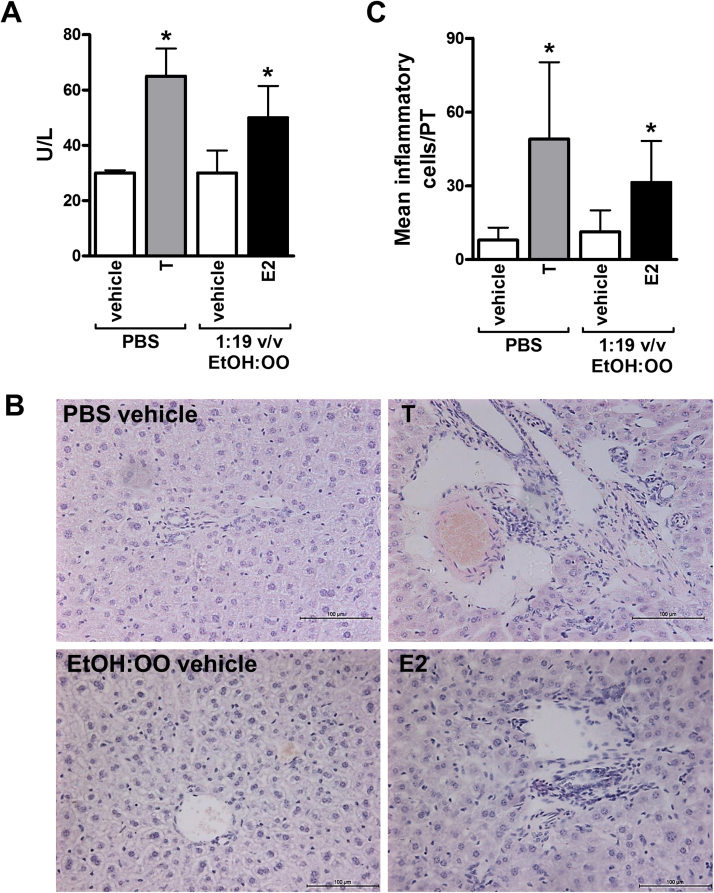

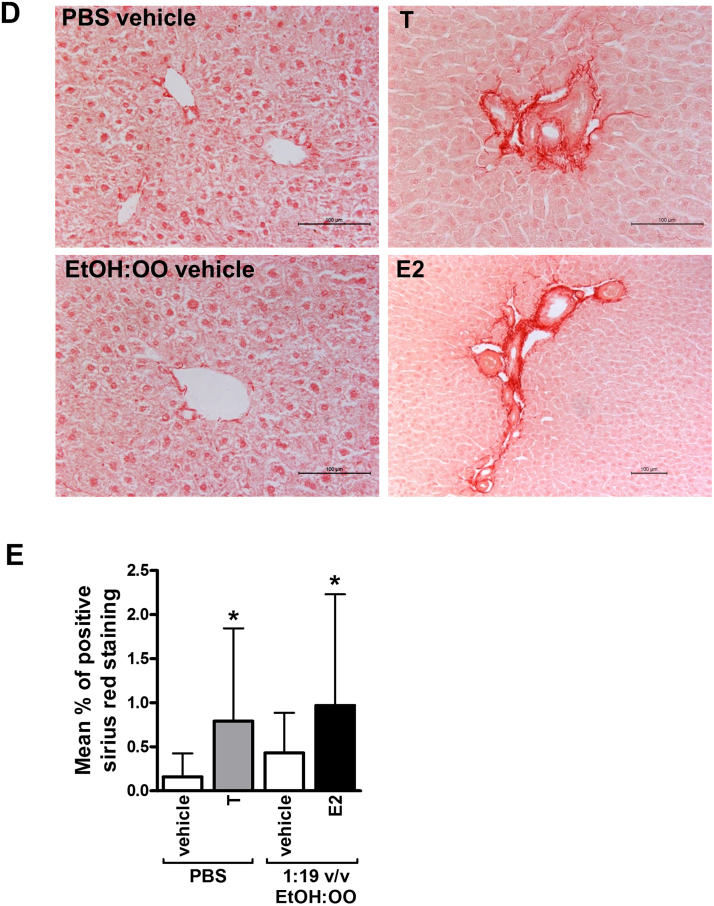
**Systemic exposure to tartrazine results in a periportal inflammatory cell recruitment in mice.** Adult male C57Bl/6 mice were administered either tartrazine (T, 4 animals), E2 (4 animals) or relevant vehicle control (PBS for tratrazine, 3 animals; 1:19 ethanol:olive oil (v/v) for E2, 4 animals) by 10 daily intraperitoneal injections over 14 days. **A**, serum ALP, *significantly different activity versus vehicle control using the Student’s *t*-test (two tailed), p > 0.95. **B**, H&E-stained liver sections from animals treated as indicated, typical views chosen. **C**, quantification of portal tract inflammatory cells, *significantly different number of portal tract cells versus vehicle control using the Student’s *t*-test (two tailed), p > 0.95 based on at least 10 randomly selected portal tracts per animal. D, sirius red-stained liver sections from animals treated as indicated, typical views chosen. E, quantification of sirius red positive stained area, *significantly different activity versus vehicle control using the Student’s *t*-test (two tailed), p > 0.95.

Fig. 1A demonstrates that there was an increase in serum alkaline phosphatase (ALP) levels following treatment with either E2 or tartrazine. Fig. 1B and C indicate that treatment with either E2 or tartrazine resulted in an increase in inflammatory cells in the portal tract region in the liver and an increase in collagen deposition (fibrosis) was observed (Fig. 1D and E).

These data suggest that direct exposure to both oestrogen (E2) and tartrazine resulted in periportal inflammation and mild fibrosis.

### 3.2 Tartrazine, its major gut-derived and endogenous metabolites and a contaminant of the food additive do not activate the murine oestrogen receptors

Despite intolerance reactions in man such as urticaria (Rajan et al., 2014), estimated to be around 0.12% of the population (Elhkim et al., 2007), data regarding the metabolism of tartrazine in experimental animals and man is limited.

Tartrazine is predominantly metabolised to sulphanilic acid (which may be further N-acetylated) and 4-sulphophenylhydrazine in the gut. These metabolites are absorbed and appear in the urine and only a minor proportion of any oral dose of tartrazine is absorbed intact (Jones et al., 1964, Roxon et al., 1966, Ryan et al., 1969a, Ryan et al., 1969b). The enzymatic azo reduction of tartrazine has been shown to be dependent on gut microorganisms (Roxon et al., 1967, Ryan et al., 1969a, Ryan et al., 1969b). After intraperitoneal administration of 2.4 mg/kg bw of 14C-tartrazine, between 64 and 96% of the dose was recovered unchanged in urine within 24 h in rats and rabbits and no other products were reported. At higher doses, free and conjugated sulphanilic acid begin to be detected in the urine (Jones et al., 1964). Based on these data, the catabolism of tartrazine is summarised in Supplementary Fig. 2.

**Fig. 2:**
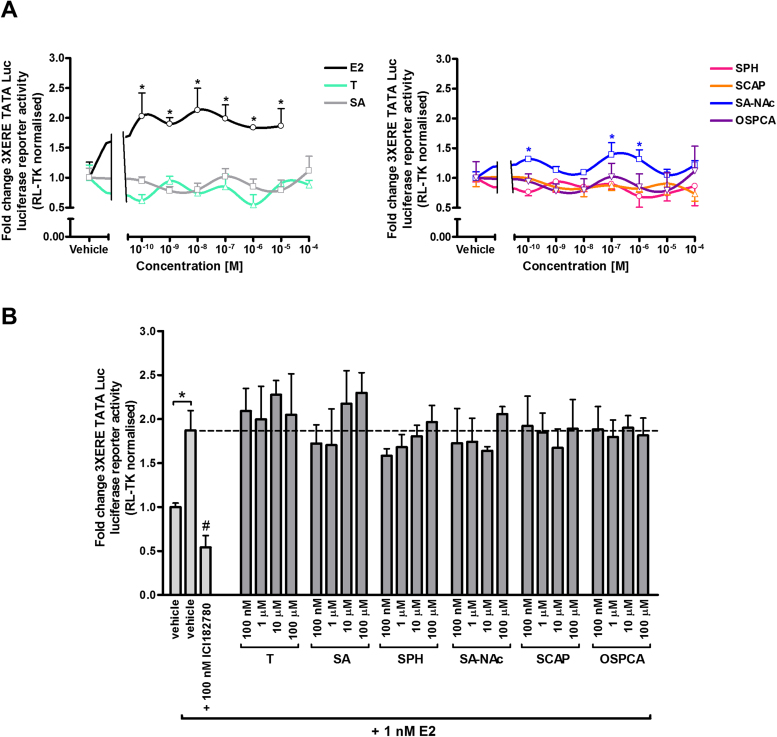
**Tartrazine, its sulphonated metabolites or major sulphonated contaminant are neither agonists nor antagonists of the mouse ERα.** Luciferase reporter gene (3XERE TATA Luc) assay in LTPA cells co-transfected with a pcDNA3.1 expression construct encoding the mERα. **A**, cells were treated with E2, tartrazine (T) or its sulphonated metabolites (SA, sulphanilic acid; SA-NAc, sulphanilic acid N-acetate; SCAP, 1-(4-sulphophenyl)-3-carboxy-4-amino-5-pyrazolone; SPH, sulphophenylhydrazine) or major sulphonated contaminant 5-oxo-1-(4-sulphophenyl)-2-pyrazoline-3-carboxylic acid (OSPCA); at the indicated concentrations for 24 h. Data are the mean and standard deviation luciferase activity from 3 separate determinations from the same experiment, typical of at least 3 separate experiments. Data are expressed as fold change versus vehicle-treated cells (vehicle: 0.1% v/v DMSO for E2 and tartrazine or 0.1% (v/v) PBS for SA, SPH, SCAP, SA-NAc and OSPCA). *Significant increase (p > 0.95) over vehicle treated cells using one-way ANOVA with Dunnett’s post-hoc test. **B**, cells were pre-treated with 100 nM ICI182780 for 6 h followed by treatment with ICI182780, tartrazine or metabolites in the presence of 1 nM E2 for 24 h. Data are the mean and standard deviation luciferase activity from 3 separate determinations from the same experiment, typical of at least 3 separate experiments and expressed in fold change versus vehicle-treated cells (vehicle: 0.1% v/v DMSO for E2 and tartrazine or 0.1% v/v PBS for SA, SPH, SCAP, SA-NAc and OSPCA). *Significant increase (p > 0.95) over cells treated with vehicle only using Student’s T-test (two-tailed). #Significant decrease (p > 0.95) over cells treated with 1 nM E2 in the absence of ICI182780 using One-way ANOVA with Dunnett’s post-hoc test.

Fig. 2A demonstrates that E2 activated the mERα, resulting in trans-activation of reporter gene expression at concentrations as low as 10pM and in a dose-dependent manner, whereas there was no evidence for trans-activation with tartrazine or its gut-derived and endogenous metabolites or a contaminant of the food additive at concentrations up to 100 μM. Fig. 2B further indicates these compounds also did not inhibit E2-dependent trans-activation of reporter gene expression, suggesting that these compounds are not ERα antagonists.

**Fig. 3:**
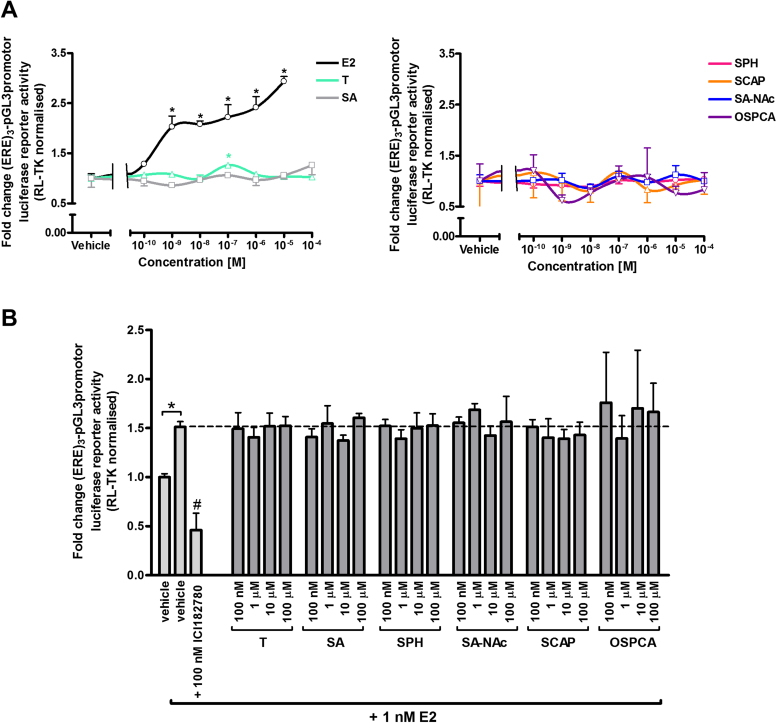
**Tartrazine, its sulphonated metabolites or major sulphonated contaminant are neither agonists nor antagonists of the mouse ERβ variant 1.** Luciferase reporter gene ((ERE)3-pGL3promotor) assay in 603B cells co-transfected with a pcDNA3.1 expression construct encoding the mouse ERβ variant 1. **A**, cells were treated with E2, tartrazine or its sulphonated metabolites or major sulphonated contaminant at the indicated concentrations for 24 h. Data are the mean and standard deviation luciferase activity from 3 separate determinations from the same experiment, typical of at least 3 separate experiments. Data are expressed in fold change versus vehicle-treated cells (vehicle: 0.1% v/v DMSO for E2 and tartrazine or 0.1% v/v PBS for SA, SPH, SCAP, SA-NAc and OSPCA). *Significant increase (p > 0.95) over cells treated with the equivalent vehicle using One-way ANOVA with Dunnett’s post-hoc test. **B**, cells were pre-treated with 100 nM ICI182780, followed by treatment with ICI182780, tartrazine, sulphonated metabolites or major sulphonated contaminant with the addition of 1 nM E2 for 24 h. Data are the mean and standard deviation luciferase activity from 3 separate determinations from the same experiment, typical of at least 3 separate experiments and expressed in fold change versus vehicle-treated cells (vehicle: 0.1% v/v DMSO for E2 and tartrazine or 0.1% v/v PBS for SA, SPH, SCAP, SA-NAc and OSPCA). *Significant increase (p > 0.95) over cells treated with vehicle only using Student’s T-test (two-tailed). #Significant decrease (p > 0.95) over cells treated with 1 nM E2 in the absence of ICI182780 using one-way ANOVA with Dunnett’s post-hoc test.

Fig. 3A shows that E2 activated the mERβv1 resulting in trans-activation of reporter gene expression at concentrations as low as 1 nM and in a dose-dependent manner, whereas there was no evidence for trans-activation with tartrazine or its gut-derived and endogenous metabolites or a contaminant of the food additive at concentrations up to 100 μM. Fig. 3B further indicates these compounds also did not inhibit E2-dependent trans-activation of reporter gene expression, suggesting that these compounds are not ERβv1 antagonists.

**Fig. 4:**
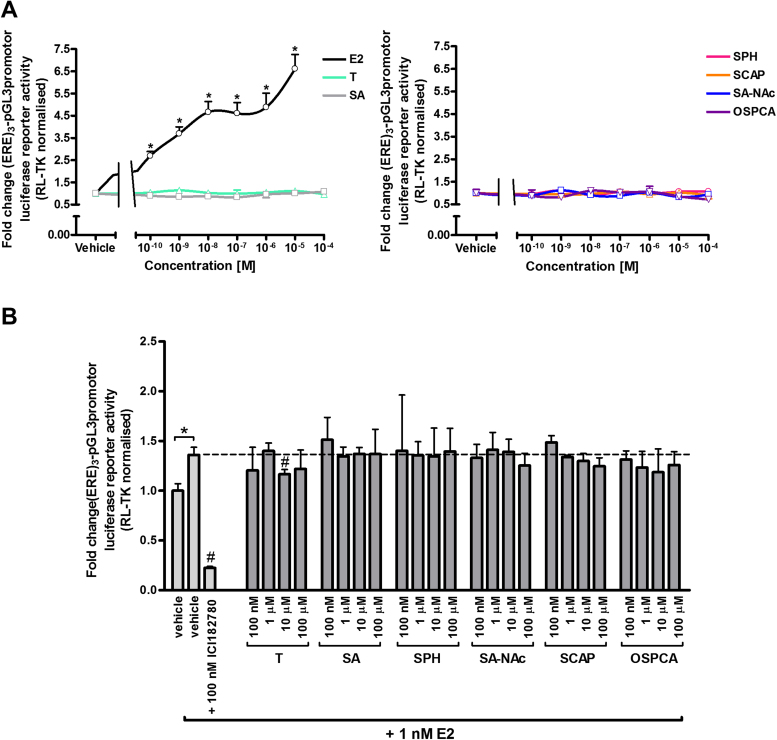
**Tartrazine, its sulphonated metabolites or major sulphonated contaminant are neither agonists nor antagonists of the mouse ERβ variant 2.** Luciferase reporter gene ((ERE)3-pGL3promotor) assay in 603B cells co-transfected with a pcDNA3.1 expression construct encoding the mouse ERβ variant 2. **A**, constitutively activate mouse ERβ variant 2 was de-activated with 100 nM ICI182780 for 6 h followed by several wash steps with sterile PBS. Cells were then treated with E2, tartrazine or its sulphonated metabolites or major sulphonated contaminant at the indicated concentrations for 24 h. Data are the mean and standard deviation luciferase activity from 3 separate determinations from the same experiment, typical of at least 3 separate experiments. Data are expressed in fold change versus vehicle-treated cells (vehicle: 0.1% v/v DMSO for E2 and tartrazine or 0.1% v/v PBS for SA, SPH, SCAP, SA-NAc and OSPCA). *Significant increase (p > 0.95) over cells treated with the equivalent vehicle using one-way ANOVA with Dunnett’s post-hoc test. **B**, cells were pre-treated with 100 nM ICI182780 for 6 h followed by treatment with ICI182780, tartrazine, sulphonated metabolites or major sulphonated contaminant with the addition of 1 nM E2 for 24 h. Data are the mean and standard deviation luciferase activity from 3 separate determinations from the same experiment, typical of at least 3 separate experiments and expressed in fold change versus vehicle-treated cells (vehicle: 0.1% v/v DMSO for E2 and tartrazine or 0.1% v/v PBS for SA, SPH, SCAP, SA-NAc and OSPCA). *Significant increase (p > 0.95) over cells treated with vehicle only using Student’s T-test (two-tailed). #Significant decrease (p < 0.95) over cells treated with 1 nM E2 in the absence of ICI182780 using one-way ANOVA with Dunnett’s post-hoc test.

Previous work by this lab has shown that the mouse ERβv2 is constitutively active and that pre-treatment with ICI182780 and wash out de-activates transcriptional activity and renders the receptor amenable to activation by subsequent exposure to oestrogens such as E2 or ethinyloestradiol (Meyer et al., 2017). Fig. 4A demonstrates that E2 activates the mERβv2 (after de-activation), resulting in trans-activation of reporter gene expression at concentrations as low as at least 100pM and in a dose-dependent manner, whereas there was no evidence for trans-activation or antagonism with tartrazine or its gut-derived and endogenous metabolites or a contaminant of the food additive at concentrations up to 100 μM. Fig. 4B further indicates that these compounds did not inhibit E2-dependent (1 nM) trans-activation of reporter gene expression, in contrast to ICI182780, indicating that tartrazine, its gut-derived and endogenous metabolites or a contaminant of the food additive are not antagonists of the mERβv2. Supplementary Fig. 3 indicates that tartrazine – but not its gut-derived and endogenous metabolites or a contaminant of the food additive – activates the human ERβ although only significantly at concentrations of tartrazine in excess of around 500 μM.

These data therefore suggest that neither tartrazine, its gut-derived and endogenous metabolites nor a contaminant of the food additive interact with the murine ERs.

### 3.3 Tartrazine administered systemically is not a mERa activator in vivo

The major biological function of oestrogens in women is to regulate the variety of physiological changes associated with female reproduction (in sexually mature women) (Bondesson et al., 2015). These changes are most physiologically overt via developmental changes in reproductive-relevant tissues such as the uterus and breast and an established assay for these effects in vivo is through determination of uterine wet weight changes on exposure to oestrogens (Reel et al., 1996).

**Fig. 5:**
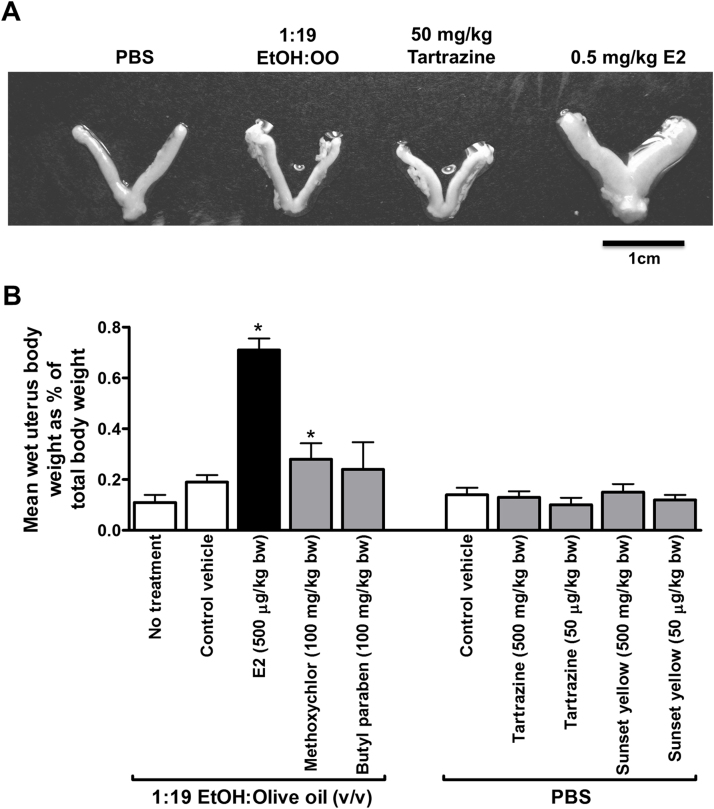
**Tartrazine lacks an uterotrophic effect in mice in vivo.** Nineteen day old female C57Bl6 mice were administered the indicated compound by single intraperitoneal injection for four consecutive days before study termination and excision of uteri on day 5. **A**, photomicrograph of typical uteri at termination demonstrating physiological effect of treatments. **B**, mean relative wet weight of uteri at study termination. Data are the mean and standard deviation of at least 4 animals/group, *significantly different wet weight versus vehicle control using the Student’s *t*-test (two tailed), p > 0.95.

Examination of mouse ERα activation in vivo by mouse uterine bioassay shows that administration of E2 and the xenooestrogen pesticide – methoxychlor – resulted in an increase in uterine wet weight. In contrast, neither dose levels of tartrazine – 0.5 mg/kg bw/day (0.067 fold of the ADI) nor 50 mg/kg bw/day (6.7 fold of the ADI) – gave rise to a change in uterine wet weight (Fig. 5A and B). Administration of sunset yellow, which was also identified as a hERα activator in vitro (Axon et al., 2012), also had no effect on uterine wet weight (Fig. 5A and B). Note that E2, methoxychlor and butylparaben were administered in an ethanol: olive oil vehicle whereas all other compounds were soluble in PBS. Moderate ethanol consumption has been shown to increase circulating oestrogen levels (Gill, 2000) and to stimulate oestrogen signalling (Fan et al., 2000) which may account for the mild oestrogenic effect of the ethanol:olive oil vehicle commonly used by others in animal studies involving steroid dosing (Sawada et al., 2000, Evans et al., 2002) versus PBS-dosed mice.

These data indicate that systemic exposure to tartrazine at levels that exceed those likely to occur through oral exposure and absorption, did not result in any observed oestrogenic effect in vivo in mice.

### 3.4 Oral exposure to tartrazine caused gut and liver inflammation without cholestatic liver injury in mice

Tartrazine is a widely used colour and exposure in man is primarily via oral exposure in food. Therefore, to determine the toxicological relevance of the hepatic effects observed with tartrazine exposure after intraperitoneal exposure to its use as a food additive, mice were orally exposed to tartrazine at 50 mg/kg bw/day for up to 10 weeks. Since tartrazine is often added to alcoholic drinks; alcohol is known to affect the gut microbiota and/or gut permeability and that these effects are a major driver of adverse hepatic effects to alcohol in animal models (Kirpich et al., 2012, Szabo, 2015, Scarpellini et al., 2016), the effects of co-exposure to alcohol and tartrazine was also examined. Mice were pre-treated with ethanol or dextrose (as a control for the energy content of ethanol) for 14 days prior to administration of tartrazine and/or ethanol for 10 weeks.

**Fig. 6:**
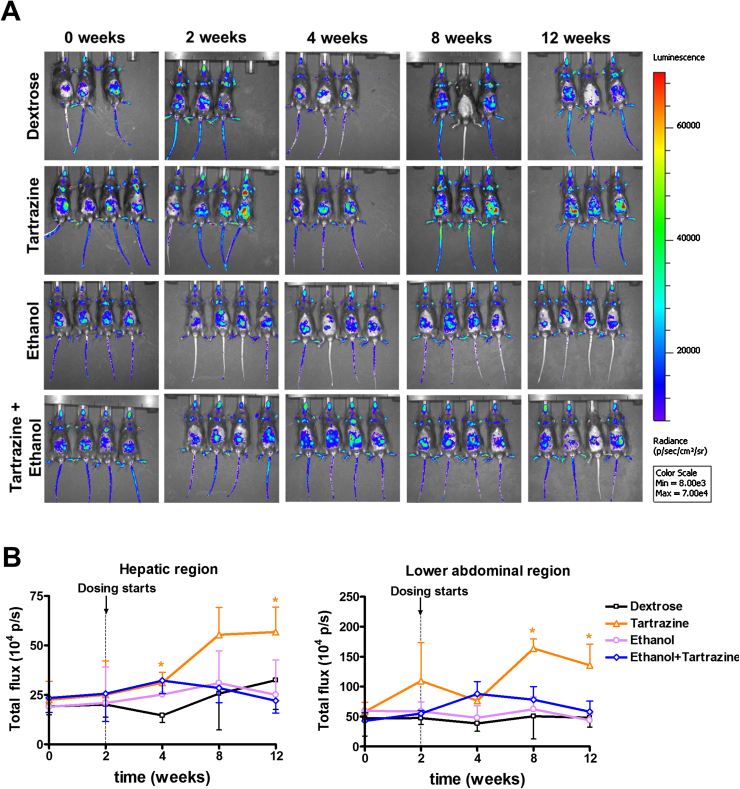

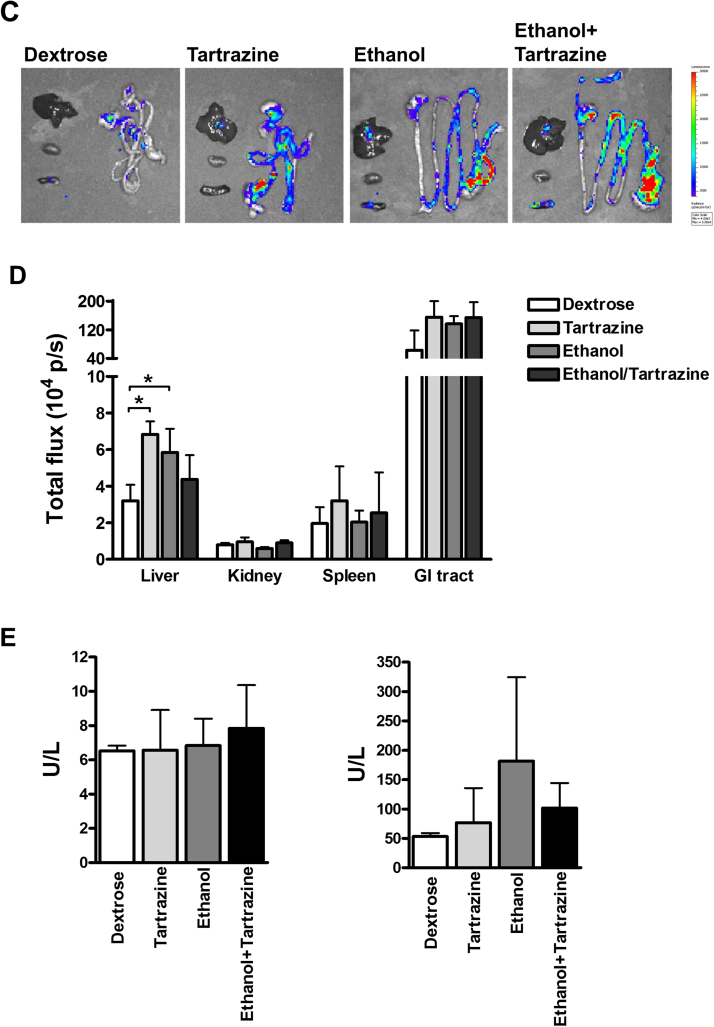
**Oral exposure to tartrazine results in gastrointestinal and hepatic inflammation without leading to cholestasis.** Adult male C57Bl/6 Tg(NF-kB) and wild type mice were initially orally administered twice daily with either ethanol or dextrose for 2 weeks and then additionally with or without tartrazine for a further 10 weeks as outlined in methods section. Mice in the control groups were dosed with ethanol or dextrose solution alone. **A**, IVIS images of Tg(NF-kB) mice at the indicated times after initial treatment. **B**, Integrated photon emission analysis of light emission profiles of the hepatic and abdominal regions of live Tg(NF-kB) animal images and the indicated times after initial treatment, data are the mean and standard deviation of at least 3 animals/group, *significantly different from control vehicle treated mice using the Student’s *t*-test, two-tailed, p > 0.95. **C**, IVIS images of Tg(NF-kB) mouse organs at termination of the study (12 weeks after study initiation). **D**, Integrated photon emission analysis of light emission profiles of the organs from Tg(NF-kB) animal images at termination of the study (12 weeks after study initiation), data are the mean and standard deviation of at least 3 animals/group, *significantly different from control vehicle treated mice using the Student’s *t*-test, two-tailed, p > 0.95. **E**, serum ALP (left panel) and ALT (right panel) at termination of the study (12 weeks after study initiation), data are the mean and standard deviation of at least 3 animals/group, *significantly different from control vehicle treated mice using the Student’s *t*-test, two-tailed, p > 0.95.

IVIS imaging of Tg mice for inflammatory responses via increases in NF-kB luciferase expression demonstrated that there was a significant increase in abdominal region inflammation (corresponding to the gastrointestinal tract) by 10 and 12 weeks of the study, and by 12 weeks in the hepatic region in response to tartrazine treatment (Fig. 6A and B). Ethanol treatment did not result in any apparent inflammatory effects and inhibited the inflammation associated with tartrazine exposure (Fig. 6A and B).

Examination of individual organs at the end of the study confirmed that the inflammation observed via live animal imaging was associated with the liver and gastrointestinal tract (Fig. 6C). Of note, the inflammation associated with the gastrointestinal tract was located at the colon (Fig. 6C). At 12 weeks, analysis of serum liver enzyme activities suggested that there was no evidence for periportal liver injury on the basis of serum ALP levels (Fig. 6D). Histological examination for inflammatory cells around the periportal regions of the liver lobule and fibrosis – as observed after treatment with oestrogens – indicated that all treatment groups were similar to control treated animals (data not shown). A three-fold increase in serum ALT compared to control animals was observed in response to ethanol treatment [although not statistically significant, is typical of the increases observed in other studies (Abdelmegeed et al., 2013, Wang et al., 2015, Zhang et al., 2016) was the only evidence of mild hepatocellular injury (Fig. 6E).

These data therefore indicate that oral administration of tartrazine at 6.7 fold the current ADI over 10 weeks resulted in a mild inflammatory response in the gut and liver, but that this was not associated with any significant liver injury. Co-treatment with ethanol reduced the inflammatory response to tartrazine.

### 3.5 Tartrazine, its major gut-derived and endogenous metabolites and a contaminant of the food additive inhibit murine hepatic sulphotransferases

**Fig. 7:**
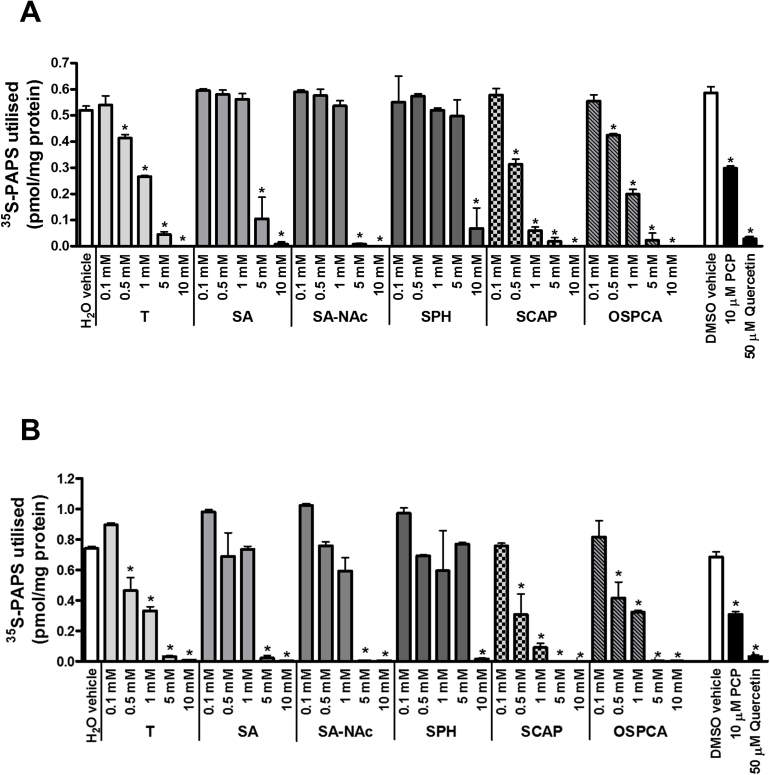
**Tartrazine, its sulphonated metabolites or major sulphonated contaminant inhibit murine hepatic dopamine sulphotransferase activity in murine S9 preparations. A**, 35S PAPS utilisation in murine hepatic S9 fraction prepared from C57Bl/6 mice. S9 fractions were incubated with 60 μM substrate dopamine (with pargyline at 1 mM to inhibit monoamine oxidases) alone (vehicle) or in combination with inhibitors PCP, quercetin or tartrazine, its gut-derived metabolites (SA, SA-NAc, SPH, SCAP) and the contaminant OSPCA at concentrations as indicated. **B**, S9 fractions were incubated with 1 μM substrate oestrone alone (vehicle) or in combination with inhibitors PCP, quercetin or tartrazine, its gut-derived metabolites (SA, SA-NAc, SPH, SCAP) and the contaminant OSPCA at concentrations as indicated. All results were normalised to protein concentration. Data are mean and standard deviation of 3 separate determinations. *Significantly different versus vehicle control using One-way ANOVA with Dunnett’s post-hoc modifications, p > 0.95.

Tartrazine has been reported to be an inhibitor of hepatic sulphotransferases using several substrates and an S9 preparation of human liver, the most potent inhibition (94 ± 3% inhibition at 6.7 μM tartrazine) observed when dopamine was the substrate (Bamforth et al., 1993). The effect of tartrazine, its sulphonated metabolites and a major sulphonated contaminant of the food additive on mouse hepatic S9 sulphation was therefore examined. Fig. 7A demonstrates that tartrazine, all 4 metabolites and the major contaminant of the food additive inhibited dopamine sulphotransferase in a dose-dependent manner in murine hepatic S9 extracts. However, significant inhibition was not seen until concentrations greater than 100 μM. SCAP and OSPCA appeared to be the most potent inhibitors of murine dopamine sulfotransferase activity with significant inhibition observed at 500 μM (Fig. 7A). Tartrazine, its sulphonated metabolites and a major sulphonated contaminant of the food additive also inhibited oestrone sulphation in a similar manner (Fig. 6B) as well as sulphation of dehydroepiandrosterone, ρ-nitrophenol or 4-methylumbelliferone in murine S9 extracts (data not shown) suggesting that the inhibitory action is via a competition with PAPS for sulphotransferases.

## 4 Discussion

Tartrazine (E 102) is a sulphonated dye used as an additive in food. Tartrazine is also used in some cosmetics and other products. It is known to elicit intolerance reactions in a small fraction of the exposed population and that sensitive individuals may react to tartrazine at dose levels within the ADI (EFSA Panel on Food Additives and Nutrient Sources added to Food (ANS), 2009).

Since tartrazine has been shown to activate the human ERα in in vitro cell model test systems by several independent labs (Datta and Lundin-Schiller, 2008, Axon et al., 2012) and oestrogens are cholestatic in vivo (Ozkan et al., 2015), we initially hypothesised that tartrazine will have cholestatic effects in vivo via an interaction with the ERα. We demonstrate in this paper that tartrazine, when administered intraperitoneally at a dose (in excess of the ADI of 7.5 mg/kg bw/day) of 50 mg/kg bw/day (10 daily doses over 14 days) resulted in periportal inflammation and mild injury suggestive of a cholestatic effect in mice. However, to confirm a cholestatic effect for tartrazine, bile flow and/or reductions in transporter-mediated efflux of bile constituents would be required. Acute, high levels of systemic oestrogen exposure (e.g. 100-fold normal circulating levels) leads to cholestatic effects in the liver, accompanied by portal tract inflammation and a fibrotic reaction (Axon et al., 2010). Intraperitoneal administration of tartrazine to mice resulted in qualitatively similar hepatic pathological effects to that seen after acute oestrogen administration, therefore we initially proposed that these effects could be associated with activation of murine ERs, alterations in hepatic transporter expression and subsequent cholestatic liver injury. However, we demonstrate that tartrazine, 4 of its sulphonated metabolites and a major sulphonated contaminant of the food additive lack murine ERα agonist or antagonist activities in in vitro cell based assays. These observations were supported by an absence of any effect on uterine growth in mice by tartrazine, in contrast to detectable increases after oestrogen or methoxychlor (a xenoestrogen insecticide) administration. Furthermore, there have been no indications for an adverse effect on reproduction or development with tartrazine (EFSA Panel on Food Additives and Nutrient Sources added to Food (ANS), 2009). In studies by Tanaka (2006) and Tanaka et al. (2008), deleterious effects on reproductive parameters were not demonstrated up to and including dose levels of 773 and 1225 mg tartrazine/kg bw/day for dietary supplementation for males and females, respectively, the highest dose levels tested.

The ability of tartrazine and its metabolites/major contaminant to interact with the related ERβ was also examined. The ERβ is expressed at low levels in normal human and rodent liver (Alvaro et al., 2004, Alvaro et al., 2006, Meyer et al., 2017). However, in a liver disease setting, the levels of ERβ expression in cholangiocytes is markedly increased and is thought to impact on the proliferation of bile ductules that occurs in cholestasis (Alvaro et al., 2006, Marzioni et al., 2012). In our hands, we also found that tartrazine, 4 of its sulphonated metabolites and a major sulphonated contaminant of the food additive lack murine ERβ variant 1 or variant 2 agonist or antagonist activities in in vitro cell based assays.

Since tartrazine, when used as a food additive, is mostly hydrolysed in the gastrointestinal tract and not significantly absorbed intact, the relevance of a hepatic effects of tartrazine after intraperitoneal administration in man is likely to be low, unless these hepatic effects can also be attributed to any metabolites of tartrazine that are absorbed. To test the food additive relevance of these observations, tartrazine was orally administered to mice and the effect of pre- and concurrent oral treatment with alcohol incorporated into the study since tartrazine may be consumed with alcohol and because alcohol may promote both gut permeability and hepatic inflammation. Although tartrazine alone induced inflammation in the colon and liver, there was no evidence of a periportal inflammatory cell recruitment and subsequent fibrosis. These data therefore indicate that oral administration of tartrazine at 6.7 fold the current ADI over 10 weeks resulted in a mild inflammatory response in the gut and liver, but that this was not associated with any significant liver injury. Co-treatment with ethanol reduced the inflammatory response to tartrazine. These effects occurred in the absence of any interaction with murine oestrogen receptors.

The pathological effects of oestrogens in the liver have been attributed to either an ERα-dependent suppression of transporter expression (Yamamoto et al., 2006) and/or stimulation of canalicular transporter endocytic internalization (Barosso et al., 2012) and/or to a saturation of hepatic oestrogen metabolism and transport that leads to a disruption in bile acid secretion or cholestasis (Stieger et al., 2000) and subsequent portal tract toxicity. Additional complexities include a role also for other signalling pathways such as GPR30 (Zucchetti et al., 2014). Since some bile acids undergo sulphation prior to secretion and excretion (Alnouti, 2009), the effects of tartrazine on hepatic sulphotransferase activities were examined. We demonstrate that tartrazine – and for the first time – 4 of its sulphonated metabolites and a major sulphonated contaminant of the food additive inhibited both dopamine and oestrone sulphotransferases in a dose-dependent manner in hepatic S9 extracts.

In man, under normal (e.g. non cholestatic) conditions, non-sulphated bile acids are sequestered within the enterohepatic circulation (Alnouti, 2009). Sulphation of bile acids at the 3 position by SULT2A1 is a major route of bile acid elimination, with the majority entering the systemic circulation and excreted by the kidneys into the urine (Alnouti, 2009). Sulphated bile acids reaching the gut are resistant to metabolism by the microbiota and eventual de-conjugation and metabolism to more toxic secondary bile acids occurs sufficiently late in its passage through the gastrointestinal tract that much of the bile acid is eliminated in the faeces. Thus, bile acid sulphation is a mechanism for bile acid elimination in man (Alnouti, 2009). The apparent inflammatory effects observed in the gastrointestinal tract and liver may be associated with a modulation of bile acid sulphation and excretion. However, given that the doses of tartrazine employed in these studies were 6.7 fold in excess of the current ADI, the absence of any periportal inflammatory cell recruitment and fibrosis effects after oral exposure, even on a background of chronic high levels of exposure to alcohol, is unlikely to be of toxicological relevance in respect of its use as a food additive. In rats and mice, sulphation of bile acids is a minor metabolic route and renal excretion of bile acids is more reliant on hydroxylation at the 6 position (Alnouti, 2009). An inhibition of sulphation by E2 or tartrazine in murine liver is therefore likely to have a less significant impact on bile acid excretion and toxicity in mice than man and therefore, the histopathological effects observed in mice after of tartrazine exposure could under-estimate the potential hepatic effects in man. Polymorphisms – including copy number variations (Schulze et al., 2013) – in human SULT2A1 are known (García-Anguita et al., 2013) may also result in increased sensitivity to tartrazine in man. There may therefore be a potential for tartrazine to alter bile homeostasis in man given its potential additional ability to activate the human ERα, although more studies will be required to determine if this could be the case.

## Conflict of interest statement

The authors declare that they have no conflict of interest.
