# Supplementary material for: Hepatic effects of tartrazine (E 102) after systemic exposure are independent of oestrogen receptor interactions in the mouse
Source: Toxicol Lett. 2017 May 5;273:55–68. doi: 10.1016/j.toxlet.2017.03.024 (PMC5429395; doi:10.1016/j.toxlet.2017.03.024)
Supplement: Supplementary file 1 [file mmc1.docx]

**Supplementary Figure 1. Analytical data for 1-(4-sulphophenyl)-3-carboxy-4-amino-5-pyrazolone (SCAP). A**, structure and general chemical and physical properties. **B**, HPLC chromatogram, major peak is SCAP. **C**, Mass spectrometry of major peak in HPLC chromatogram having a molecular mass of 300 Da. D, 1H-NMR in D2O.

**Supplementary Figure 2. Metabolic profile of tartrazine.** The catabolism of tartrazine in rodents is summarised, based on Jones et al., 1964; Jones et al., 1964; Roxon et al., 1966; Roxon et al., 1967; Ryan et al., 1969a,b.

**Supplementary Figure 3. Tartrazine – but not its gut-derived and endogenous metabolites or a contaminant of the food additive – activates the human ERβ.** Luciferase reporter gene ((ERE)3-pGL3promotor) assay in HEK293 cells co-transfected with a pcDNA Flag ERβ expression construct encoding a flag tagged human ERβ and RL-TK.  Cells were treated with tartrazine or its sulphonated metabolites or major sulphonated contaminant at the indicated concentrations for 24 hours. Data are the mean and standard deviation luciferase activity from 3 separate determinations from the same experiment, typical of at least 3 separate experiments. Data are expressed in fold change versus vehicle-treated cells (vehicle: 0.1% v/v DMSO for E2 and tartrazine or 0.1% v/v PBS for SA, SPH, SCAP, SA-NAc and OSPCA). *Significant increase (p > 0.95) over cells treated with the equivalent vehicle using One-way ANOVA with Dunnett’s post-hoc test. E2 activated the receptor maximally at 1nM to give a fold 1.8 fold increase in reporter gene expression versus vehicle control (data not included).
