# Supplementary figures and images for: Hepatic effects of tartrazine (E 102) after systemic exposure are independent of oestrogen receptor interactions in the mouse
Source: Toxicol Lett. 2017 May 5;273:55–68. doi: 10.1016/j.toxlet.2017.03.024 (PMC5429395; doi:10.1016/j.toxlet.2017.03.024)

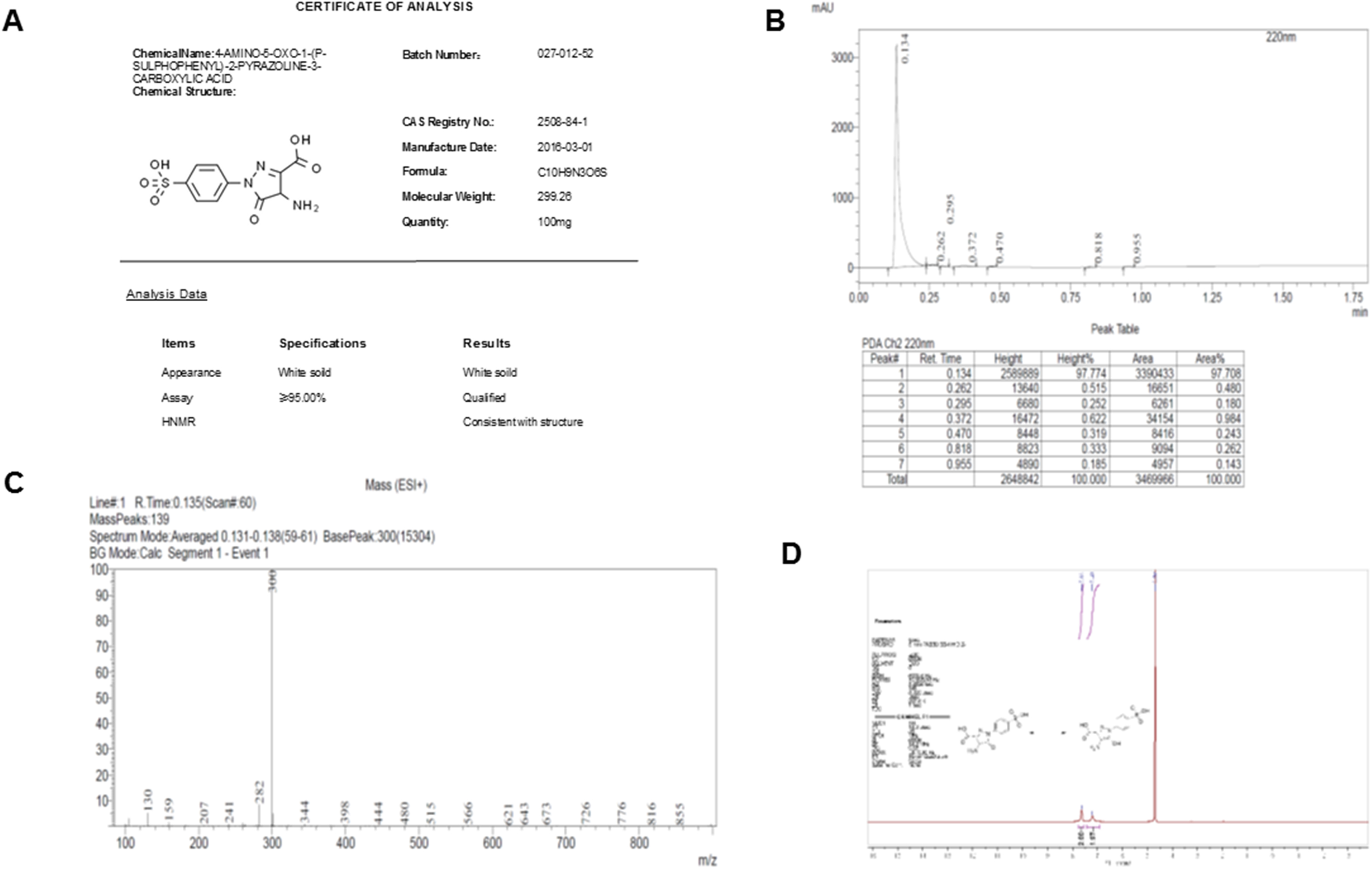

Supplement: Supplementary file 2 [file mmc2.jpg]

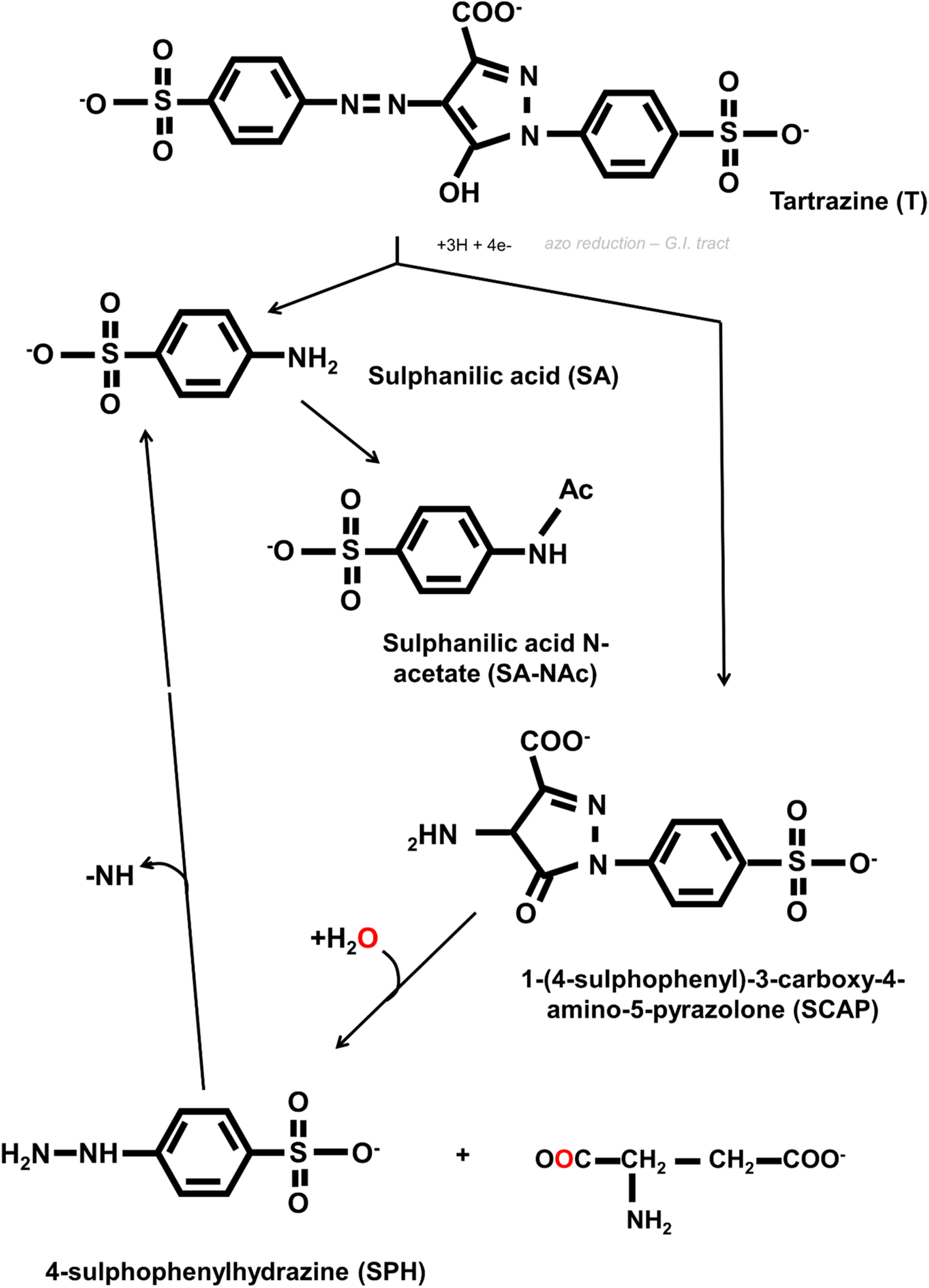

Supplement: Supplementary file 3 [file mmc3.jpg]

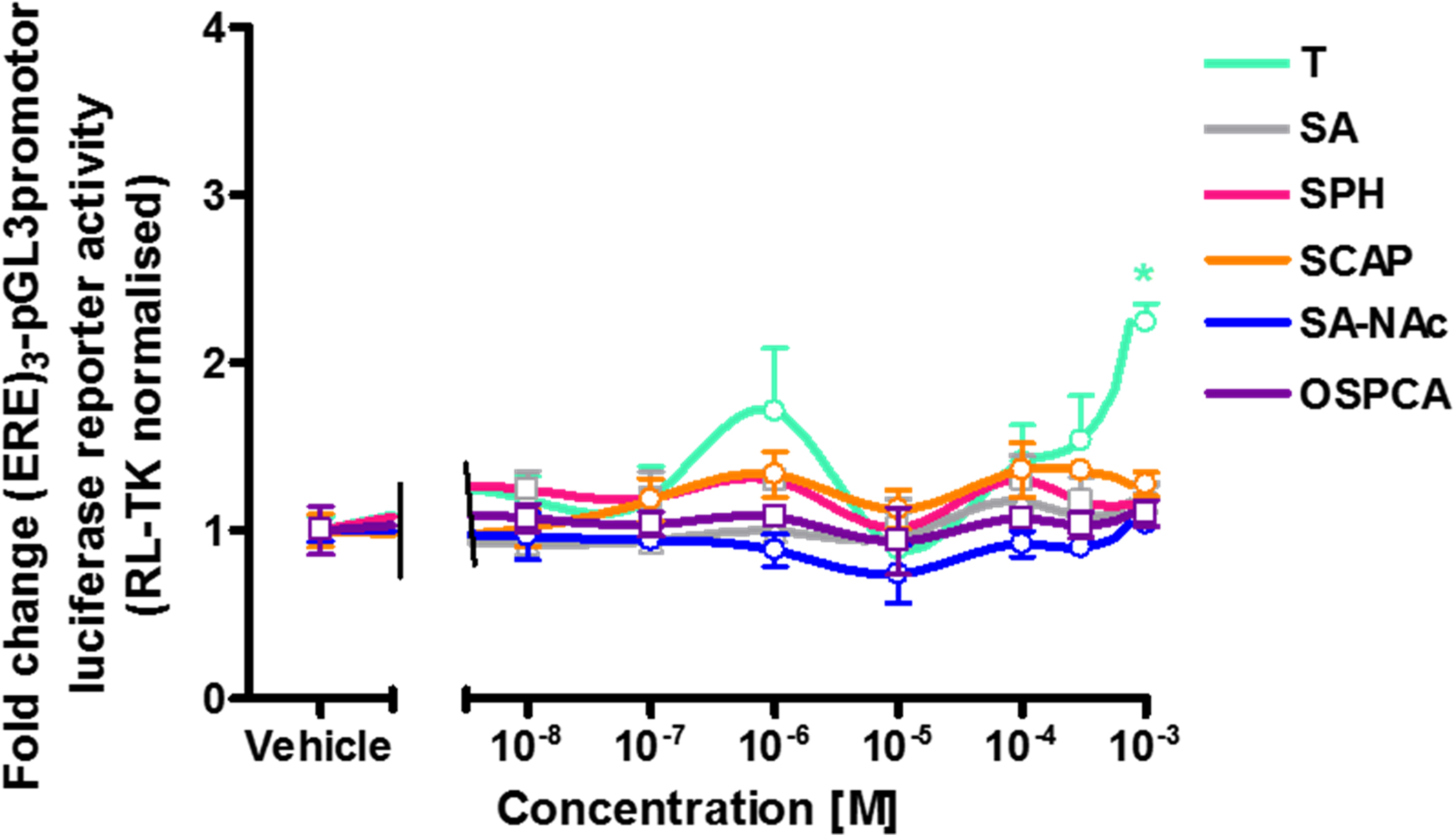

Supplement: Supplementary file 4 [file mmc4.jpg]
